# Primary Blast Traumatic Brain Injury in the Rat: Relating Diffusion Tensor Imaging and Behavior

**DOI:** 10.3389/fneur.2013.00154

**Published:** 2013-10-14

**Authors:** Matthew D. Budde, Alok Shah, Michael McCrea, William E. Cullinan, Frank A. Pintar, Brian D. Stemper

**Affiliations:** ^1^Department of Neurosurgery, Medical College of Wisconsin, Milwaukee, WI, USA; ^2^Clement J. Zablocki Veterans Affairs Medical Center, Milwaukee, WI, USA; ^3^Department of Biomedical Sciences, College of Health and Sciences, Marquette University, Milwaukee, WI, USA

**Keywords:** traumatic brain injury, blast neurotrauma, memory dysfunction, diffusion tensor imaging, magnetic resonance imaging

## Abstract

The incidence of traumatic brain injury (TBI) among military personnel is at its highest point in U.S. history. Experimental animal models of blast have provided a wealth of insight into blast injury. The mechanisms of neurotrauma caused by blast, however, are still under debate. Specifically, it is unclear whether the blast shockwave in the absence of head motion is sufficient to induce brain trauma. In this study, the consequences of blast injury were investigated in a rat model of primary blast TBI. Animals were exposed to blast shockwaves with peak reflected overpressures of either 100 or 450 kPa (39 and 110 kPa incident pressure, respectively) and subsequently underwent a battery of behavioral tests. Diffusion tensor imaging (DTI), a promising method to detect blast injury in humans, was performed on fixed brains to detect and visualize the spatial dependence of blast injury. Blast TBI caused significant deficits in memory function as evidenced by the Morris Water Maze, but limited emotional deficits as evidenced by the Open Field Test and Elevated Plus Maze. Fractional anisotropy, a metric derived from DTI, revealed significant brain abnormalities in blast-exposed animals. A significant relationship between memory deficits and brain microstructure was evident in the hippocampus, consistent with its role in memory function. The results provide fundamental insight into the neurological consequences of blast TBI, including the evolution of injury during the sub-acute phase and the spatially dependent pattern of injury. The relationship between memory dysfunction and microstructural brain abnormalities may provide insight into the persistent cognitive difficulties experienced by soldiers exposed to blast neurotrauma and may be important to guide therapeutic and rehabilitative efforts.

## Introduction

The incidence of traumatic brain injury (TBI) among military personnel in modern combat is the highest of any conflict in U.S. history (1, 2). Up to 20% of combat veterans met the criteria for TBI on post-deployment screening (3), and exposure to blasts from improvised explosive devices contributed to the unprecedented rate of mild TBI (mTBI). The overwhelming majority of events were categorized as mTBI (4), typically characterized by a consistent clinical scenario of postconcussive symptoms, cognitive dysfunction, and other functional impairments that follow a gradual course of recovery during the initial days and weeks after injury (5, 6). However, increasing evidence suggests that even mild head trauma may result in postconcussive symptoms and comorbidities many months after mTBI (7–10). Moreover, even a single blast episode may have long-term pathogenic potential and cause enduring neurodegeneration (11). Understanding the neurological consequences of blast neurotrauma is essential to enable prevention and guide therapeutic and rehabilitative efforts.

Despite the considerable number of experimental investigations of blast TBI using rodent models reported in recent years, the mechanisms underlying blast injury and the factors affecting outcomes remain unclear. Whole-body or head-only blast paradigms have highlighted the potential for different mechanisms of brain injury following blast exposure (12, 13). Likewise, the effects of the blast shockwave alone (primary injury) may be different than those involving rapid head acceleration subsequent to blast (tertiary injury) and were recently compared directly (14, 15). Understanding the time-course of recovery from blast neurotrauma is also vital to relate injury mechanisms with patient outcomes. Behavioral assessments conducted at acute (12, 16–19), sub-acute (20, 21), and chronic time points (13, 20, 21) indicate prolonged neurological abnormalities following blast. However, given the differences in injury protocols and other experimental variations between studies conducted at a single timepoint after injury, assessments performed at several time points would enable a better characterization of the time-course of deficits.

Another important factor to understand injury tolerance and clinical outcome is the effect of blast magnitude (i.e., shockwave overpressure). A number of experimental protocols incorporated differing overpressure severities to examine the physiological consequences of higher magnitude exposures (22–27), but fewer studies have assessed whether higher magnitude overpressures lead to greater behavioral deficits. This is important for the quantification of injury tolerance and may also relate to the duration of the recovery curve. While some studies demonstrated greater mortality rates at higher overpressures (18), a lack of a consistent relationship between overpressure and cognitive ability (19, 28, 29), or neuromotor performance (18) has frequently been noted. A recent study by Cernak et al. demonstrated dose dependency in Rotarod and Open Field Test (OFT) outcomes for mice exposed to whole-body 103 and 190 kPa shockwaves (13). However, a need exists to elucidate the dose-response effects and temporal recovery of behavioral outcomes following primary blast shockwave exposure, given somewhat conflicting outcomes from previous investigations.

Expanded efforts to screen military personnel for potential exposure to mTBI have addressed the growing burden of head trauma in combat. However, there is still no definitive marker of mTBI, which has hampered clinical decision-making. Non-invasive imaging techniques, particularly magnetic resonance imaging (MRI), have considerably advanced the detection and understanding of subtle brain changes following mTBI. Diffusion tensor imaging (DTI), an advanced MRI technique, is a promising method for non-invasively examining the effects of TBI. DTI probes the molecular motion of water within living tissues to infer the presence of microscopic structural abnormalities. DTI has uncovered changes in veterans exposed to blast TBI (30–32) and mTBI through other physical (non-blast) mechanisms (33, 34). However, despite its promise, many questions remain regarding the use of DTI as a diagnostic marker. While experimental models of blast TBI were essential in identifying the physiological, metabolic, and pathological consequences of blast TBI, DTI has been infrequently applied to experimental animal models of blast under well-controlled laboratory conditions that would provide fundamental insight into the neurotrauma caused by blast forces.

The objective of this work was to examine the behavioral changes from acute to sub-acute time points, examine the dependence of behavioral outcomes on different levels of shockwave overpressures, and quantify structural damage to brain tissues using DTI following exposure to blast. Importantly, the study employed a head-only primary blast injury paradigm to provide fundamental insight into the mechanisms of blast shockwave neurotrauma and its associated behavioral consequences.

## Materials and Methods

### Shock tube apparatus

**Figure 1:**
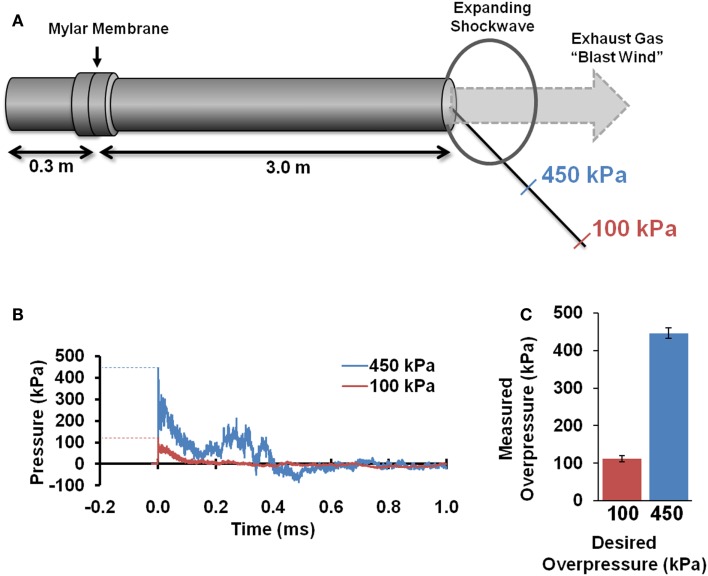
**Blast tube characterization**. A shocktube **(A)** pressurized with helium delivered blast shockwaves. Rats were placed at specified distances from the shocktube opening based on initial characterization and were placed off-axis to avoid blast wind. Pressure sensors depict the typical reflected pressures **(B)** experienced during the blast shockwave. Blast peak overpressures realized during actual exposures to the rat head **(C)** were highly accurate and reproducible (*n* = 8 for each).

A custom shock tube (Figure 1A) with a 3.6-cm inner diameter, 3.0-m driven section, and 0.3-m driver section was used to create shockwaves with different overpressure magnitudes. A mylar membrane separated driver and driven sections. The driver section was pressurized with helium until membrane rupture. The shock tube was previously characterized and was shown to produce accurate and repeatable shockwaves across a range of shockwave overpressure magnitudes (35). Pressure transducers (Piezotronics, Inc., Depew, NY, USA) were oriented to record face-on (reflected) pressures at a sampling rate of 5 MHz at selected locations and were placed immediately adjacent to the head for all blast exposures. Additional characterization of the blast pressures was confirmed by placing sensors at identical locations in the absence of a rat, and sensors were oriented to record either face-on (reflected) or side-on (incident) pressures.

### Animal procedures

All animal procedures were approved by the Institutional Animal Care and Use Committee (IACUC) at our institution. All animals were allowed access to food and water *ad libitum* before and after shockwave exposure. A 2 × 2 factorial design was employed with blast magnitude and post-injury duration as independent variables. Animals were exposed to a single blast shockwave with either a 100 or 450 kPa reflected peak overpressure (Figure 1B), hereafter referred to the low-blast and high-blast conditions, respectively. Assessments were conducted at acute (1–4 days post injury) or chronic (28–31 days post injury) time points, hereafter referred to as the 4- and 30-days post-injury (DPI) groups, respectively. Each experimental group consisted of 9–14 rats. A separate cohort of eight animals served as sham controls and underwent identical procedures without exposure to shockwave.

**Figure 2:**
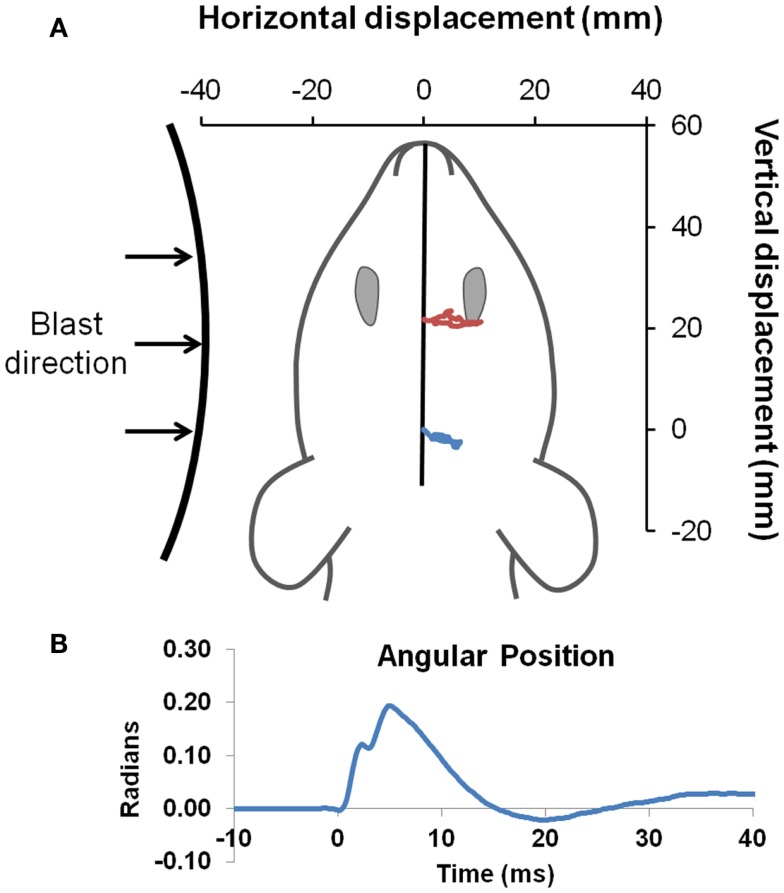
**Head kinematics during blast**. Blast was directed to the left side of all animals **(A)**. Landmarks of ink placed between the eyes (red), and on the head (blue) were tracked with high-speed video to obtain head position (drawn to scale) during exposure to the blast wave, with an example of the high-blast shown. Angular position **(B)** was derived by intersection of a line passing through the two points compared to the initial position of the head, and demonstrated only limited head motion.

On the day of injury, Sprague-Dawley rats were anesthetized with 4% isoflurane in oxygen and placed in a nose cone for continuous delivery of 1.5% isoflurane. Rats were placed 39 and 17 cm from the shock tube opening for the low- and high-blast shockwave exposure conditions, respectively (Figure 1A). Since the exhaust gasses, or blast wind, can lead to considerable head acceleration, animals were placed 40° and 20° lateral to the shock tube axis to limit blast wind exposure for the low and high-blast conditions, respectively. Prior characterization of the shock tube demonstrated minimal blast wind effects at the locations chosen for this study (35). Moreover, the head was constrained laterally and inferiorly to prevent head rotational acceleration-induced injury, and all shockwave exposures were conducted with the rat head perpendicular to the radial axis from the shock tube opening (Figure 2A). A metal cylinder was also placed around the body to limit shockwave overpressure exposure of the torso. Prior work indicated that the cylinder was effective at reducing peak overpressures to <20% of overpressure magnitudes recorded at the head. Following shockwave exposure, animals were removed from anesthesia and allowed to breathe freely. Animals were continuously monitored until return of the righting reflex, returned to their cages for recovery and observation for at least 15 min, and periodically monitored for 6 h post injury.

Following completion of the behavioral testing protocol, animals were euthanized with an overdose of pentobarbital and perfused through the left cardiac ventricle with 250 ml of phosphate buffered saline (PBS) followed by 250 ml of 4% paraformaldehyde in PBS. Brains were excised from the skull and placed in fixative for 48 h followed by PBS for long-term storage.

### High-speed videography

High-speed videography was used to record head motion during shockwave exposure. The digital video camera (Integrated Design Tools Inc., Tallahassee, FL, USA) was placed directly overhead, perpendicular to the horizontal plane of the animal, to monitor head motion at 6000 frames per second. Physical markings were placed at the center of the head (i.e., lambda landmark) and between the eyes (slightly anterior to the bregma). Video tracking software (Tracker 4.75, Open Source Physics) was used to track displacement of markings and derive head linear and rotational motion time histories. Angular position data were digitally filtered using a lowpass Butterworth filter with a 500-Hz cutoff frequency followed by numerical differentiation to derive angular velocity and acceleration.

### Morris water maze

The Morris water maze (MWM) Visuo-Spatial Learning Paradigm (36, 37) quantified post-traumatic anterograde amnesia and spatial learning. The MWM consisted of a 10 cm diameter platform submerged 1 cm below the surface of a 25 cm deep pool of water. Pool diameter was 183 cm. Three testing sessions were conducted over three consecutive days. Each session consisted of eight separate trials wherein rats were initially placed at each of the four cardinal locations (N, E, S, W). During each trial, rats were allowed to swim until finding and mounting the hidden platform or until 60 s had passed. The location platform was randomized between sessions but was consistent within the same session, and the first trial of each session was excluded from analysis. Visual cues, including those internal to the maze and external cues in the room, were maintained between sessions. A computerized tracking system and software (Ethovision V8.0, Noldus Information Technology, Wageningen, The Netherlands) recorded movement of the rats within the maze. Latency to find the hidden platform and number of unsuccessful trials were computed from these recordings. Spatial learning deficits manifest as greater latencies and a higher number of unsuccessful trials.

### Elevated plus maze

The elevated plus maze (EPM) assessment is an ethologically relevant test that quantified anxiety-related behaviors. The maze consisted of four perpendicular 10 cm × 90 cm arms connected by a 10 cm × 10 cm central platform. One pair of opposing arms was enclosed by 40-cm high walls, while the other two arms and the center platform were uncovered. The entire apparatus was composed of black plexiglass and was raised 50 cm above the floor. Rats were initially placed on the central platform facing one of the open arms and allowed to explore the maze for 5 min. A camera mounted above the maze and Ethovision software automatically quantified the number of arm changes, time spent in the open arms, and the number of head dips. Behaviors associated with increased anxiety included decreased time in the open arms and fewer head dips.

### Open field test

The OFT was used to examine anxiety and spontaneous exploratory behavior. The apparatus consisted of a flat, 100-cm diameter circular arena with 20-cm walls. Illumination was provided by overhead lights. Rats were initially placed in the center and allowed to explore the arena for 5 min. The center of the arena consisted of a 66-cm diameter circle, with the remainder considered the border region. The distinction was indicated within the software and did not include any physical markings. An overhead mounted camera along with Ethovision software automatically quantified measures including total distance traveled and total time spent in the center of the maze. Behaviors associated with increased anxiety include a decreased time spent in the center.

### Statistical analysis

A two-factor Analysis of Variance (ANOVA) was used to identify statistically significant interactions for behavioral metrics between blast severity and post-injury time for groups exposed to blast, followed by *post hoc* tests when warranted. In the absence of a significant interaction, a one-way ANOVA was conducted with the sham group included to test for a significant main effect of either blast magnitude or time after injury using separate models. *Post hoc* comparisons were performed if the overall test was significant. For all tests, significance was set at a *p*-value of <0.05.

### Magnetic resonance imaging

A 9.4 T horizontal bore Bruker BioSpec was employed for MRI procedures. Fixed brains were immersed in susceptibility-matching fluid (Fomblin; Solvay Solexis, NJ, USA) and placed in a custom 20-mm diameter inductively coupled loop-gap coil. Room temperature was maintained throughout the experiment. A series of gradient echo scout images were acquired to ensure reproducible slice orientations and placement within the magnet. A spin-echo sequence (TR = 2000 ms, TE = 21 ms) with Stejskal–Tanner pulsed gradients was used to perform diffusion-weighting along 12 non-orthogonal directions at a *b*-value of 1200 s/mm^2^ and two non-diffusion weighted images (*b* = 0 s/mm^2^). A double-echo spin-echo readout (echo spacing = 3.3 ms) was used to improve signal to noise ratio (SNR) by magnitude averaging of the individual echo images. Twenty axial slices at a thickness of 0.5 mm and an in-plane resolution of 0.2 mm^2^ covered the entire brain and brainstem. The entire MRI experiment was completed in approximately 1 h.

### MRI data analysis

Diffusion weighted images were corrected for eddy current distortions, and the diffusion tensor was calculated on voxel-by-voxel basis using fMRI Software Library (FSL). For spatial registration, a study-specific template was created using tensor-based registration implemented in the DTI-TK software package (38). An iterative procedure using rigid body, affine, and diffeomorphic registration was used to create a template at a final resolution of 200 μm^3^. Tensor volumes were spatially smoothed with an anisotropic three-dimensional filter implemented in Matlab (39). Maps of fractional anisotropy (FA) were derived and used for subsequent statistical analysis.

Differences between blast-exposed groups and the sham group were assessed using a two-sample *t*-test. Permutation-based inference testing implemented in the FSL was used for statistical hypothesis testing, and voxels with *p* < 0.05 (uncorrected for multiple comparisons) and clusters >100 voxels were considered statistically significant. Relationships between FA and behavioral tests were examined using linear regression analysis of the blast-exposed animals. For each of the behavioral tests (MWM, EPM, and OFT), behavioral outcomes were regressed against FA values on a voxel-by-voxel basis to identify voxels with a significant *F*-statistic (*p* < 0.05).

A complementary region of interest (ROI) analysis was performed to assess distributed FA abnormalities. The mean FA map from all animals (*n* = 60) was segmented into gray and white matter using a threshold of 0.25. The ROIs were manually edited to include only the cerebral white matter tracts and cerebral cortex by excluding the cerebellum, brainstem, and subcortical structures, and each hemisphere was assessed separately. The FA map from each animal was converted to z-scores (*z* = (*x−*μ)/σ) on a voxel-by-voxel basis where *x* is the individual FA value, μ and σ are the mean and standard deviation FA value of the sham group, respectively. To ensure robustness, an additional six blast sham animals were included (*n* = 14). Moreover, a leave-one-out approach was used for the sham group by calculating the *z*-scores for each of the individual brains compared to the mean and standard deviation of the remaining 13. The percentage of voxels exceeding a *z*-score of ±2 was calculated separately for the WM and GM ROIs (31, 34). The resulting quantitative metric, percentage of abnormal FA voxels, was compared to the sham group for each of the blast-exposed groups using a Student’s *t*-test and a Bonferroni correction for multiple comparisons.

### Histology

Fixed brains were processed for routine paraffin embedding and cut at a thickness of 4 μm. Selected sections were stained in 0.01% toluidine blue O for 5 min and mounted. Alternatively, sections for immunofluorescent staining were placed in 10 mM sodium citrate buffer in PBS, microwaved for 5 min, and allowed to cool to room temperature. Sections were incubated at 4°C overnight with primary antibodies for glial fibrillary acidic protein (GFAP; 1:500; EMD Millipore, Billerica, MA, USA) or cleaved caspase-3 (1:500, EMD Millipore, Billerica, MA, USA). Incubation with the secondary antibody (Alexa Fluor; 1:500, Molecular Probes) was performed for 30 min at room temperature. Sections were mounted and imaged with a Nikon microscope.

## Results

### Blast shockwave and TBI model characterization

Pressure time histories depict the standard experimental approximation of the Friedlander waveform of the primary shockwave overpressure (Figure 1B). Peak reflected overpressures measured at the rat head had a high accuracy and reproducibility, with measured values of 451 ± 25 (*n* = 21) and 112 ± 14 (*n* = 22) kPa for the 450 and 100 kPa conditions, respectively (Figure 1C). Positive durations were 0.34 ± 0.15 ms for the high-blast exposures and 0.46 ± 0.21 ms for the low-blast exposures. Pressures measured near the rat thorax were considerably lower at 63 ± 26 and 19 ± 5 kPa, respectively. To ensure the presence of the rat did not alter blast pressure measurements, separate blasts were performed with sensors placed in the location of the rat head but without a rat in position. Measurements demonstrated similar reflected pressures of 450 ± 23 kPa (*n* = 3) and incident pressures of 110 ± 5.6 kPa (*n* = 6). The experimental apparatus also limited head motion. Using high-speed video analysis, a maximum lateral displacement of 6.98 mm was measured in the high-blast condition, with an angular acceleration of <150 krad/s^2^ (Figure 2B). Minimal head motion was noted for the low-blast condition with an acceleration of <10 krad/s^2^. Therefore, any behavioral deficits or structural brain damage resulting from these exposures was most likely the result of shockwave passage through the brain tissues and its subsequent effects rather than head rotational acceleration.

### Memory deficits in shockwave exposure groups

**Figure 3:**
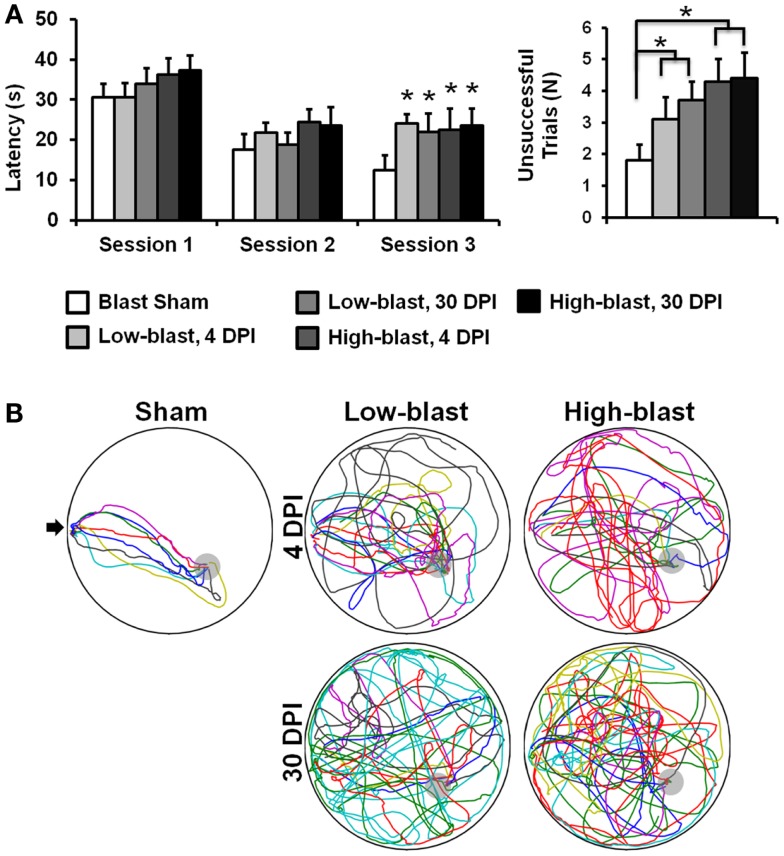
**Morris water maze**. Memory and learning deficits were evident in the blast-exposed animals compared to sham animals **(A)**. Swim path traces from the final trial **(B)**, with each animal shown in a different color, reveal the difficulties in finding the hidden platform (gray) in the blast-exposed animals compared to the sham animals. The entry point is indicated with an arrow. Error bars indicate SEM. *p < 0.05.

On the MWM Visuo-Spatial Learning Paradigm, all experimental groups demonstrated the ability to learn (Figure 3A), since latency to find a hidden platform decreased over time (*p* < 0.001). On the first day of testing, there were no significant differences in latency between the groups (*p* = 0.18). On the second day, a main effect of blast severity approached, but did not meet the threshold for significance (*p* = 0.06). On the third day, the main effect of blast severity was significant (*p* = 0.034). Both the low-blast (*p* = 0.015) and high-blast (*p* = 0.016) groups had significantly greater latencies to find the platform than the sham group (Figure 3B), but the two groups were not significantly different than one another (*p* = 0.97). Time after blast had no effect on latency between the 4 and 30 DPI groups (*p* = 0.86), indicating persistent cognitive deficits following shockwave exposure.

The number of unsuccessful trials across all 18 sessions of the MWM also indicated impaired memory and cognition in animals exposed to blast (Figure 3A). There was no significant interaction between blast severity and time (*p* = 0.73), but a significant main effect of blast severity was evident (*p* < 0.019). The high-blast group had significantly more unsuccessful trials compared to the sham blast group (*p* = 0.006), whereas the low-blast group was not significantly different from animals exposed to either high-blast (*p* = 0.075) or sham (*p* = 0.15) blast. The main effect of time after injury for the number of unsuccessful trials was not significant (*p* = 0.18). Time spent in the target quadrant had a significant interaction between blast severity and time after injury (*p* = 0.04). In the *post hoc* analysis, the low-blast group at 30 DPI spent significantly more time in the target quadrant than the high-blast groups at either 4 (*p* = 0.029) or 30 (*p* = 0.01) DPI. No other individual groups were significantly different from one another. A main effect of blast severity was also significant (*p* = 0.01), with the low-blast group spending more time in the target quadrant than the high-blast (*p* = 0.008) or sham (*p* = 0.031) groups. All other comparisons for time in the target quadrant were not significant.

### Open field assessment

**Figure 4:**
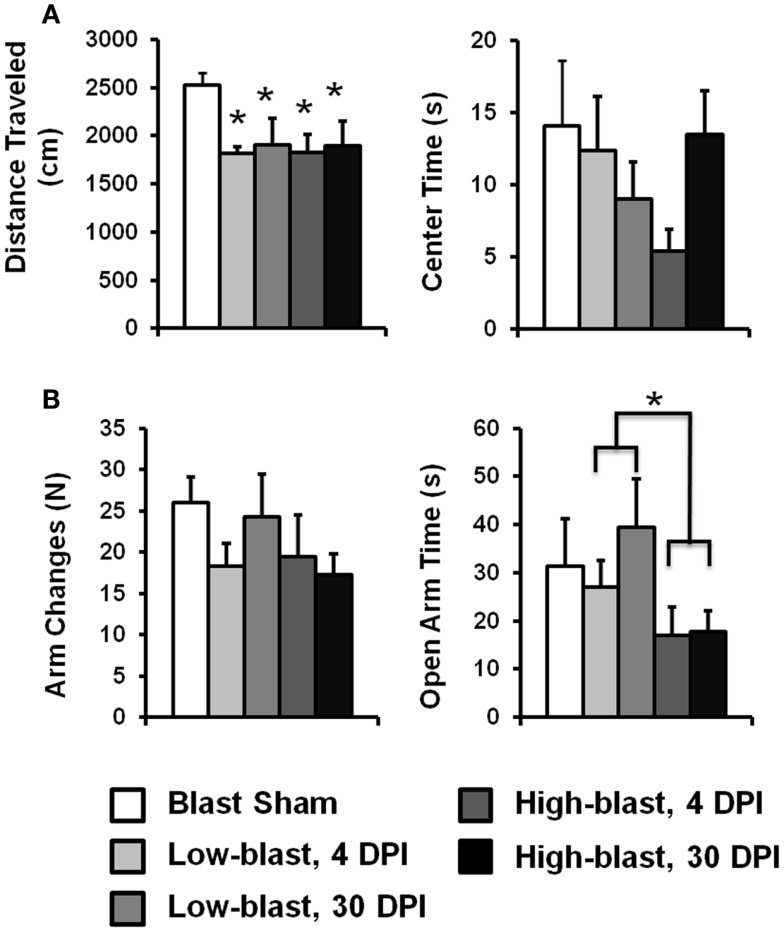
**Open field test and elevated plus maze**. Emotionality and anxiety was examined with the Open Field Test **(A)** and the Elevated Plus Maze **(B)** for each of the four blast conditions and sham group. Error bars indicate SEM. *p < 0.05.

The OFT was used to assess locomotor activity and anxiety behavior (Figure 4A). Activity, as measured by the total distance traveled during the test, did not have a significant interaction between blast severity and time after injury (*p* = 0.69). A significant main effect of blast severity was observed (*p* = 0.029). Compared to the sham blast group, animals exposed to low (*p* = 0.018) or high (*p* = 0.011) blast conditions had significantly less activity, but they were not significantly different from one another (*p* = 0.76). The effect of time after injury was not significant (*p* = 0.84). Likewise, the amount of time spent in the center of the OFT, a measure of anxiety, did not have a significant interaction between blast severity and time after injury (*p* = 0.20). Time in the center was also not significant for either the main effect of blast severity (*p* = 0.51) or time after injury (*p* = 0.63).

### Emotionality changes in shockwave exposure groups

Exploratory behavior on the EPM served as an indicator of emotionality. The amount of time spent in the open arms of the EPM (Figure 4B), a metric associated with anxiety, did not have a significant interaction with blast severity and timing after injury (*p* = 0.39). However, the main effect of blast severity was significant (*p* = 0.037). The high-blast group spent significantly less time in the open arms compared to the low-blast (*p* = 0.013) group. The high-blast (*p* = 0.11) and low-blast (*p* = 0.71) groups were not significantly different from the sham group. The main effect of time after injury was not significant (*p* = 0.19).

The number of arm changes in the EPM did not have a significant interaction between blast severity and time after injury (*p* = 0.63). The main effect of blast severity was not significant (*p* = 0.35), although the number of arm changes was decreased by 26 and 29% in the low and high-blast groups, respectively (Figure 4B). The main effect of timing after injury was not significant (*p* = 0.98).

### Shockwave exposure caused spatially dependent and progressive DTI abnormalities

**Figure 5:**
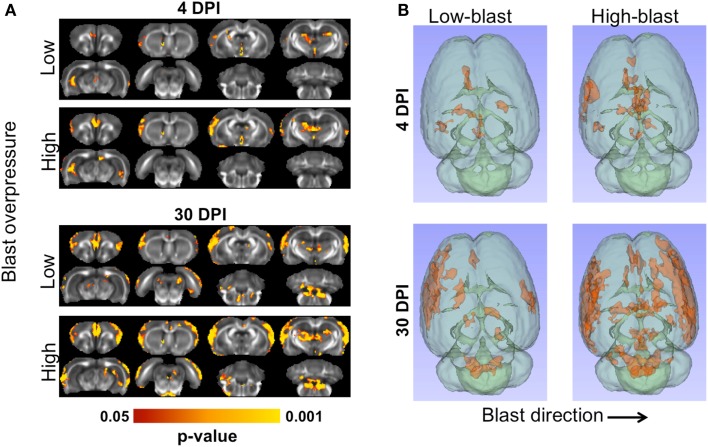
**Microstructural abnormalities following primary blast**. The brains of animals exposed to blast shockwave underwent DTI and compared to animals exposed to sham blast **(A)**. Significant FA decreases were evident at 4 (left) and 30 days post-blast (right). At both timepoints, greater numbers of significant voxels were evident in the animals exposed to high-blast compared to those at exposed to low-blast. Compared to 4 days post-blast, there were more significant voxels at 30 days post-blast in both blast exposures. Decreased FA was most prominent in the cortex, but also included the thalamus, and ipsilateral ventral hippocampus. The three-dimensional distribution of significant changes compared to sham are shown **(B)** as viewed from the top of the brain. The direction of blast is indicated by the arrow and is identical for both panels.

Shockwave exposure caused significant changes in FA. A distinct pattern of abnormalities emerged related to blast severity and time after injury, with significant differences between each of the blast-exposed groups compared to the sham group (Figure 5A). At 4 DPI, significant decreases in FA were evident in the ipsilateral cortex, medial prefrontal cortex (mPFC), hippocampus, and thalamus and were more pronounced in the animals exposed to high-blast than those exposed to low-blast shockwaves. At 30 DPI, significant decreases in FA were evident in both blast exposures. Brain regions affected at 30 DPI included those affected at 4 DPI along with additional injury evident in the contralateral cortex and brainstem. The voxelwise statistical tests were uncorrected for multiple comparisons to aid in visualizing the spatial pattern of FA changes across all of the blast-exposed groups with the same sham group used for all comparisons. The decreases in FA were greater and more widespread in the group exposed to high-blast compared to those exposed to low-blast. The three-dimensional visualization of significant differences are shown in Figure 5B. The effect sizes (Cohen’s D) for the significant clusters in each of the groups compared to the sham group were 0.417 and 0.407 for the low-blast and high-blast at 4 DPI, respectively, and 0.493 and 0.495 for the low-blast and high-blast groups, respectively, at 30 DPI. When corrected for multiple comparisons at *p* < 0.05 by controlling the familywise error, the voxelwise differences did not survive and were therefore followed up by an unbiased ROI analysis.

**Figure 6:**
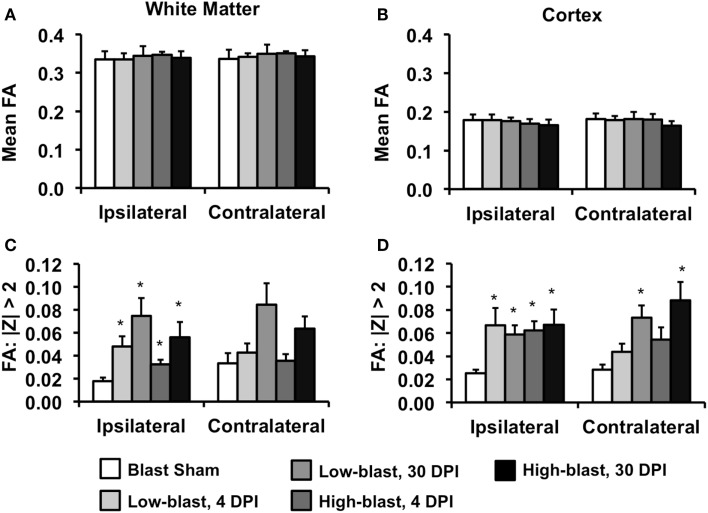
**Region of interest DTI analysis**. The mean FA of the white matter tracts **(A)** or cerebral cortex **(B)**, assessed separately for the ipsilateral and contralateral hemispheres, were not significantly different between any of the groups. In contrast, the percentage of voxels with FA values >2 standard deviations from the mean (*z*-scores) compared to the sham group revealed significant group differences. In the white matter **(C)** no significant differences were evident in either the ipsilateral or contralateral hemispheres. In the cerebral cortex **(D)**, the percentage of abnormal voxels ipsilateral to the blast exposure was significantly greater in all blast-exposed groups compared to the sham group. In the contralateral hemisphere, only groups 30 days post-blast had a significantly greater percentage of abnormal FA voxel compared to the sham group. Error bars indicate standard deviation.*p < 0.05 compared to sham.

A ROI analysis was performed to complement the voxelwise findings and quantitatively examine the laterality of the DTI changes (Figure 6). ROIs encompassing the left (ipsilateral) or right (contralateral) cerebral white matter (Figure 6A) or cortical gray matter (Figure 6B) revealed that mean FA was not significantly different between any of the groups. However, an alternative method quantifying the number of abnormal FA voxels for each animal relative to a spatially aligned sham group (*z*-score) revealed significant differences between groups. In the white matter, the percentage of abnormal (|*z*| > 2) voxels was significantly greater in all of the blast-exposed groups compared to shams in the ipsilateral white matter, with greater changes observed at 30 DPI (Figure 6C). In the contralateral white matter, no significant differences were observed. However, in the cerebral cortex (Figure 6D), the number of abnormal FA voxels was significantly greater in the ipsilateral cortex for all blast-exposed groups compared to the sham group (all *p* < 0.05, corrected for multiple comparisons). In the contralateral hemisphere, the low-blast (*p* = 0.0009) and high-blast (*p* = 0.0005) exposure groups at the chronic time points were significantly different than the sham group, whereas no significant different differences were noted at the acute time point. In general, the ROI results confirmed the previous voxelwise observations. To identify whether the FA changes were predominantly associated with changes in axial diffusivity or radial diffusivity, these values were obtained from the abnormal FA voxels from each animals and averaged across groups. There were no significant differences in AD or RD between any of the groups compared to the sham group.

### Relationship between fractional anisotropy and behavior

**Figure 7:**
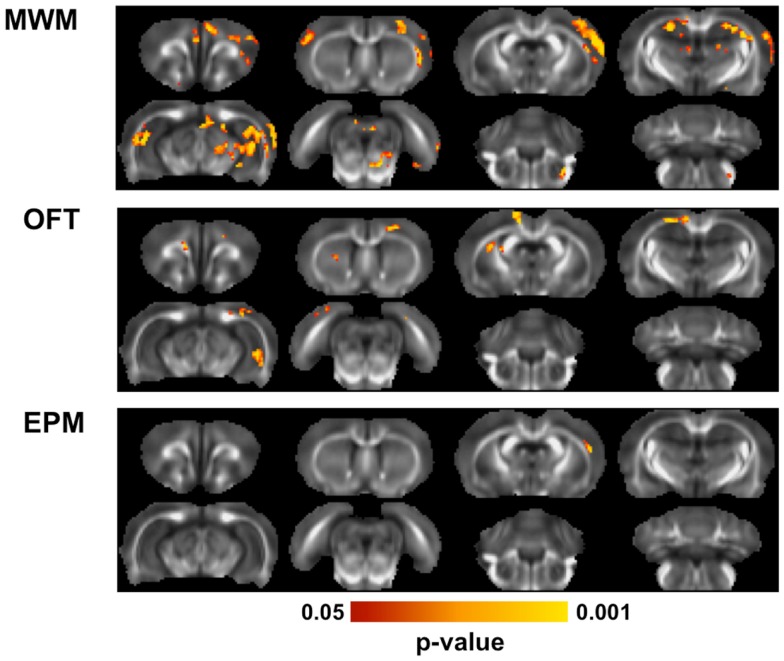
**Diffusion tensor imaging correlations with behavior**. Across all animals exposed to blast, multiple linear regression was used to determine whether behavioral outcomes were associated with brain microstructure. For each behavioral test, the outcome measures obtained from it were regressed against the FA value from all animals using a *F*-test, controlling for blast severity and DPI. In the MWM, FA was significantly related to behavioral performance in the hippocampus and portions of the motor cortices. In the OFT, a region of the ventral hippocampus and region of the external capsule were significantly related to the behavioral performance. The EPM was related to FA in only a small region of the contralateral cortex.

Linear regression analysis was performed to identify relationships between behavioral outcomes and microstructure assessed with DTI (Figure 7). In the MWM, clusters of voxels with significant linear relationships between FA and behavioral outcomes were evident bilaterally in the hippocampus, predominantly in the dorsal region. A significant region was also evident in the motor cortex, predominantly in the contralateral cortex. Across all significant voxels, target quadrant percentage was a better predictor of FA (*p* < 0.001) than number of unsuccessful trials (*p* = 0.045) or latency (*p* = 0.088). In the OFT, outcomes were significantly related to FA of the contralateral, ventral hippocampus, and a small portion of the ipsilateral external capsule. FA in these regions was significantly associated with both outcomes of the OFT, time in the center (*p* < 0.001), and distance traveled (*p* < 0.001). In the EPM, only a small region of significance was located in the contralateral cortex. Neither the number of arm changes (*p* = 0.082) or time spent in the open arms (*p* = 0.30) were independent predictors of FA in this region.

### Histological evidence of injury

**Figure 8:**
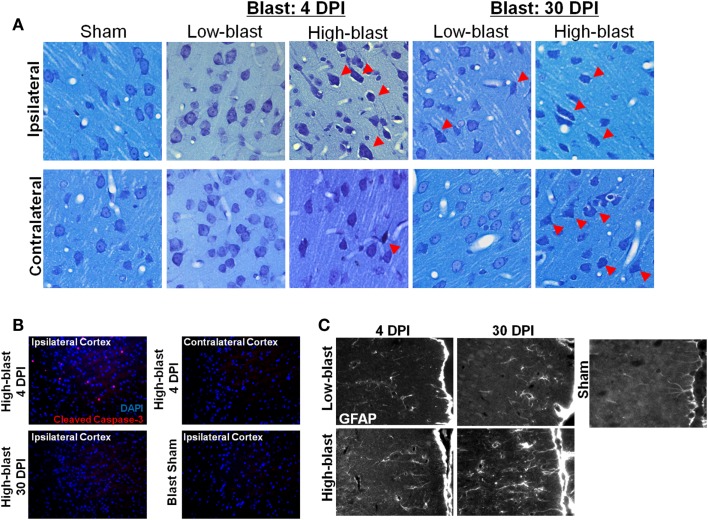
**Histological evidence of blast effects**. Brain sections from the regions exhibiting DTI changes, approximately +2.0 mm Bregma, were stained with toluidine blue **(A)**. Sections revealed numerous “dark” neurons in the brains exposed to high-blast **(C)** at both 4 and 30 days post-blast, particularly in the ipsilateral cortex, but fewer in the contralateral cortex. Only sporadic abnormal neurons were observed in the low-blast and sham brains. In a brain exposed to high-blast, staining for cleaved caspase-3 **(B)** was observed in the ipsilateral cortex at 4 days post-blast, but not in the contralateral cortex, and the stain was no longer present at 30 days post-blast. No staining was present in the sham brain. In the medial prefrontal cortex, a region with significant FA changes, astrocytes stained with GFAP exhibited extensive hypertrophy in the high-blast brain at 30 days compared to the sham brain, but astrogliosis was noticeably less at 4 days post-blast or in the low-blast condition.

No hemorrhage was evident in any of the animals exposed to shockwaves based on visual inspection of the brain surface. A qualitative analysis revealed greater astrocyte hypertrophy in the mPFC (Figure 8), a region exhibiting significant DTI abnormalities (Figure 4A), following high-blast shockwave exposure compared to low-blast shockwave exposure. A similarly greater hypertrophy was evident at chronic compared to acute time points. Correspondingly, sections stained with Toluidine blue revealed chromalytic neurons in the cortex of brains exposed to high-blast shockwaves at both acute and chronic time points, whereas the brain exposed to low-blast shockwaves contained only sparse darkly stained neurons. Staining for cleaved caspase-3, a nuclear marker of apoptosis, revealed numerous apoptotic cells within the ipsilateral cortex, whereas no apoptotic nuclei were evident in the contralateral cortex or at 30 days post-blast.

## Discussion

Animal brain injury models have proven essential to understand the consequences of blast injury. Although the precise mechanisms of blast neurotrauma are the subject of considerable investigation and debate, several important experimental factors may have a considerable influence on the findings. The current investigation employed an experimental rodent model designed to be a translational paradigm representative of human blast injury dynamics. In our animal model, the body of each rat was protected, so only the head was exposed to shockwaves. This protective approach is consistent with the setting of ballistic personal protective body armor worn by military personnel being effective at attenuating blast overpressure exposure to the thorax (40). Field injuries are more likely attributable to shockwave exposure of the head, and injury tolerances are different between shockwave exposures to the head and brain tissue compared to those involving pulmonary injury. The scaling of shockwave characteristics from the rat to the human is another important experimental concern. Construction of explosive devices using artillery shells (41) has demonstrated overpressure durations typically <10 ms. Scaling ratios presented by Bass et al. (42), albeit for pulmonary exposures, can be used to relate overpressure durations from rodent models to human-equivalent values. In the current study, scaled overpressure durations were consistent with human exposures of 2–3 ms. Isolating the effects of blast shockwave from head acceleration is an important concept to understand the mechanisms of blast injury. In the current study, the head was laterally supported during shockwave exposure, which prevented appreciable head excursion during the low-blast exposures and limited excursions during high-blast exposures. Angular accelerations were well below the threshold for mild concussion in the rat (43). Similarly, positioning rats outside and off-axis from the shock tube limited exposure to venting gas (i.e., blast wind). While exposure outside of the tube introduces shockwave dynamics such as diffraction and weakening, all rats were exposed to a repeatable shockwave profile (Figure 1) without blast wind confirmed by measured pressure profiles. Collectively, the resulting behavioral, imaging, and histological consequences of blast injury were attributable to the primary shockwave exposure and not pulmonary or rotational acceleration mechanisms.

Primary blast injury caused significant memory deficits in shockwave-exposed rats compared to sham-exposed animals, with more pronounced deficits in the higher shockwave exposure group. These data provide preliminary support for the theoretical assumption of a “dose effect” associated with more severe or repetitive blast TBI exposure. The observed memory and cognitive deficits were generally consistent with findings from other experimental investigations of shockwave-induced TBI in rodents (21, 44), although the magnitude of blast exposure differences between the studies may have implications regarding injury tolerance to blast. Goldstein and colleagues (14) demonstrated significant cognitive deficits after 77 kPa exposures, but the high magnitude head rotational accelerations potentially caused injury through various mechanisms. The cognitive deficits in the present protocol, which incorporated head restraint, were directly attributable to the interaction of the shockwave and the head. A notable difference between the present study and previous investigations is the presence of significant cognitive deficits acutely after injury. Increased latency to find the platform and greater numbers of unsuccessful trials were evident for shockwave-exposed groups at <4 days post injury. Other studies have reported no differences between controls and shockwave-exposed rats at 3–8 days post injury (21, 44, 45). The disparity could be related to the use of the Barnes Maze assessment in other studies, compared to the use of the Morris Water Maze in the current study, since the two assessments may have different sensitivities. These differences may also be attributable to differences in the injury protocol such as the used of head restraints and torso protection.

Neurobehavioral and emotional changes assessed using the EPM and OFT were also generally consistent with previous reports (44). Elevated levels of anxiety were evident for shockwave-exposed rats, with some indication of a dose response and transient symptoms. Sham rats demonstrated the greatest magnitude of open arm time, with the groups exposed to blast spending progressively less time in the open arms at the acute time point. Likewise, open field center time decreased in the sham group compared to the groups exposed to blast. These findings indicate higher levels of anxiety in more severely injured rats, again supporting a dose effect of blast TBI. This type of dose dependency for emotional changes following shockwave injury may have clinical implications for the understanding of patient condition and outcomes. Whether emotional changes are transient or persistent following shockwave exposure has been mixed. In the current study, the time in the center of the open field was decreased in the high-blast group acutely, but recovered by 1 month post injury. However, open arm time in EPM decreased acutely in the high-blast group and did not resolve by 1 month post injury. Similar behavioral paradigms at other laboratories have observed progressive emotional changes at 44 DPI (20), but no deficits at 66 DPI. Conversely, persistent decreases in open arm time from 48 h to 1 month post injury have also been observed (44). Emotional deficits have also been shown to persist up to 6 months post-injury (46). The disparities may be related to the interaction between stress and emotional dysfunction (44, 47) and require further investigation, particularly in relation to the development of PTSD in military personnel and returning veterans exposed to blast during combat (2, 4, 7).

The finding of DTI abnormalities and their spatial distribution provides some insight into the mechanisms of injury following shockwave exposure. At acute time points, decreased FA in the superficial cortex ipsilateral to the shockwave suggests a local effect of the shockwave on the brain tissue, and is consistent with previous histological studies demonstrating gliosis in the lateral (14) and medial prefrontal cortices (44). The effects may be related to high shear strain or microcavitation at the interface between the brain and cerebrospinal fluid shown in simulations of blasts in the human (48) and rat brain (27). In the current study, injuries progressed from acute to chronic time points and involved greater regions of the cortex as well as portions of the brainstem, suggesting an ongoing response similar to that observed elsewhere (49). The injury in the superficial cortex could potentially be related to the acute disruption of the blood-brain barrier (17) and it sequelae. Indeed, gliosis is a prominent pathology after blast (15) and hypertrophic astrocytes were evident in the mPFC in the current study (Figure 7). White matter injury was also evident in the current study (Figure 4), consistent with previous histological reports of axonal injury in the brainstem and cerebellum (50). Furthermore, the regions of injury could be associated with auditory regions in the brain (51). Further studies will be necessary to understand the complex relationship between biomechanical and other consequences of blast injury.

The current study employed voxelwise analysis of FA images since the orientation of the blast was consistent across all animals. However, since blast neurotrauma in humans is highly variable with respect to intensity, direction, and other factors, alternative approaches to capture the heterogeneity have been proposed. Comparing an individual to a control group to derive group-averaged measures (31, 34) or single subject maps (52) of abnormal FA was shown to have greater sensitivity to TBI than measures of mean FA. In the current study, this approach was used to quantify the voxelwise changes. As expected, there were no differences in whole brain mean FA values between any of the groups. However, quantifying the number of abnormal FA voxels in each subject relative to the control group and group-averaging this metric was sensitive to group differences (Figure 6). The results were largely consistent with the voxelwise findings, but also provide greater insight into the directionality of the FA changes, since this is conflicting in human DTI studies following TBI. In the cortex, the FA decrease was greater with higher blast intensity and with longer durations from the injury. In contrast, FA increased in the white matter acutely and appeared to normalize at the 30 day timepoint. This is the opposite that of the white matter FA decreases observed in severe TBI (53). In human mTBI, however, increased white matter FA was often observed in the acute (54) and semi-acute (34) periods whereas decreased FA was consistently observed in the more chronic phases (30–32). Thus, although FA is a sensitive biomarker of injury following TBI, interpreting the changes in FA are complex with respect to the underlying pathology (55).

The relationship between DTI, cognitive, and neurobehavioral abnormalities identifies a putative link between regional brain abnormalities and the resulting clinical manifestations of blast injury. While the current findings were observed in an experimental animal model, they likely have significant translational relevance to our understanding of the pathophysiology and clinical effects of blast injury in human. Impairment in memory function was related to abnormalities in the hippocampus (Figure 7), as expected although the precise mechanism for the changes is not clear. A quantitative assessment of the biological basis of the DTI findings would provide greater insight into the injuries evident in humans after blast. One limitation of the current study is the use of fixed tissues. Fixation has been shown to maintain anisotropy (56), but *in vivo* measurements would enable a better appreciation of longitudinal changes following TBI. On the other hand, imaging of fixed tissues affords substantial improvements in image quality and resolution compared to *in vivo* and thereby better sensitivity to subtle changes. Another limitation is that blast injuries were performed at a single orientation relative to the shock tube (side-on). The importance of orientation has been demonstrated using simulations (27, 48) and experimental studies (19, 57), but a whole brain assessment such as that provided by DTI has yet to be performed. Utilization of serial, *in vivo* MRI will be important to fully characterize the effects of blast TBI and the evolution from acute to chronic injury.

## Conclusion

In a rat model of blast TBI, primary blast exposure was sufficient to induce cognitive and neurobehavioral deficits and microstructural injury detected with DTI. Importantly, both outcomes scaled with the magnitude of the blast shockwave, reflecting a dose effect that has implications to the fundamental pathophysiology and neurologic sequelae of blast TBI in humans. Moreover, the correlation between neurological deficits and DTI findings underscores the connection between brain injury and behavior that has particular relevance to advancing the science of therapeutic interventions and prevention of long-term effects of blast injury in military service members and veterans.

## Conflict of Interest Statement

The authors declare that the research was conducted in the absence of any commercial or financial relationships that could be construed as a potential conflict of interest.
